# Photovoltaic Performance of Dye-Sensitized Solar Cells Containing ZnO Microrods

**DOI:** 10.3390/nano9121645

**Published:** 2019-11-20

**Authors:** Seong Il Cho, Hye Kyeong Sung, Sang-Ju Lee, Wook Hyun Kim, Dae-Hwan Kim, Yoon Soo Han

**Affiliations:** 1 School of Advanced Materials and Chemical Engineering, Daegu Catholic University, Gyeongbuk 38430, Korea; pokw1231@naver.com; 2 Department of Organic Material Science and Engineering, Pusan National University, Busan 46241, Korea; amrm1995@naver.com; 3 Division of Energy Technology, Daegu Gyeongbuk Institute of Science and Technology (DGIST), Daegu 42988, Korea; tglee@dgist.ac.kr (S.-J.L.); kwh1980@dgist.ac.kr (W.H.K.)

**Keywords:** dye-sensitized solar cell, ZnO, nanoflowers, microrods

## Abstract

At an elevated temperature of 90 °C, a chemical bath deposition using an aqueous solution of Zn(NO_3_)_2_·6H_2_O and (CH_2_)_6_N_4_ resulted in the formation of both nanoflowers and microrods of ZnO on F-doped SnO_2_ glass with a seed layer. The nanoflowers and microrods were sensitized with dyes for application to the photoelectrodes of dye-sensitized solar cells (DSSCs). By extending the growth time of ZnO, the formation of nanoflowers was reduced and the formation of microrods favored. As the growth time was increased from 4 to 6 and then to 8 h, the open circuit voltage (*V_oc_*) values of the DSSCs were increased, whilst the short circuit current (*J_sc_*) values varied only slightly. Changes in the dye-loading amount, dark current, and electrochemical impedance were monitored and they revealed that the increase in *V_oc_* was found to be due to a retardation of the charge recombination between photoinjected electrons and I_3_^−^ ions and resulted from a reduction in the surface area of ZnO microrods. A reduced surface area decreased the dye contents adsorbed on the ZnO microrods, and thereby decreased the light harvesting efficiency (LHE). An increase in the electron collection efficiency attributed to the suppressed charge recombination counteracted the decreased LHE, resulting in comparable *J_sc_* values regardless of the growth time.

## 1. Introduction

Photovoltaic cells convert solar energy into direct current electricity using p-type and n-type semiconductors. Traditionally, solid-state junction devices, usually made of silicon, have dominated this field, drawing on the experience and material availability arising from the semiconductor industry. The dominance of inorganic solid-state junction cells is being challenged by the advent of third-generation photovoltaic cells such as dye-sensitized solar cells (DSSCs), polymer solar cells, and perovskite solar cells [1,2,3]. DSSCs based on nanoporous TiO_2_ films have been extensively researched due to their comparable photovoltaic efficiency, potentially low production cost, low toxicity, and rapid manufacturing process. In addition, the advantages of DSSCs for real-world applications, including transparent and multicolor options, or both, easy architectural integration, and short energy payback time have drawn attention. 

The general DSSC consists of a dye-adsorbed semiconductor electrode (i.e., TiO_2_ photoelectrode), an electrolyte, and a Pt-coated counter electrode [1,4]. The TiO_2_ photoelectrodes in the cells play an important role in the photovoltaic efficiency. Highly porous TiO_2_ films provide extended surface area for dye adsorption resulting in the enhanced light harvesting efficiency (LHE) of DSSCs. TiO_2_, as an n-type semiconductor, is also a good transporter of the electrons injected from the photoexcited dyes. As another n-type semiconductor, ZnO has a band gap energy of 3.3 eV, similar to that of TiO_2_ (3.2 eV), and has much higher electron diffusivity than TiO_2_. It also has a high electron mobility of 115–155 cm^2^ V^−1^ s^−1^, which is favorable for effective electron transport through the ZnO layer and for reduction of the charge recombination rate [5,6]. In addition, the diverse morphologies of ZnO including nanorods, nanowires, nanosheets, nanobolts, or nanoflowers allows for various designs of photoelectrodes for ZnO based DSSCs. The diverse morphology of ZnO is attributable to the anisotropic growth of ZnO, unlike the crystalline structure of TiO_2_ [5,6,7,8]. ZnO is thus considered to be a viable alternative to TiO_2_ in DSSCs.

The reported power conversion efficiencies (PCEs) of ZnO-based DSSCs range from under 1% to the highest value of 7.5% [5,6,9], mainly dependent on the deposition condition and the morphology of nano-structured ZnO. Extensive research on improving the PCE of DSSCs based on the nanostructure of ZnO has been conducted since the first report on dye-sensitized ZnO [10], but little attention has been given to the use of ZnO microrods. To the best of our knowledge, DSSCs with ZnO microrods as photoelectrodes have never been reported. In this study, ZnO microrods grown on F-doped SnO_2_ (FTO) glass were prepared through a chemical bath deposition. Dye-sensitized microrods were utilized as the photoelectrodes of DSSCs and their photovoltaic performance was examined. 

## 2. Materials and Methods 

### 2.1. Materials

Commercial FTO (sheet resistance ~7 Ω/square) glass (TCO22-7), N719 dye (Ruthenizer 535-bisTBA), hot-melt adhesive (SX1170-60PF, Surlyn), and iodide-based electrolytes (AN-50) were purchased from Solaronix. Zinc acetate dehydrate [Zn(CH_3_COO)_2_·2H_2_O], zinc nitrate hexahydrate [Zn(NO_3_)_2_·6H_2_O], and hexamethylenetetramine [HMTA, (CH_2_)N_4_] (Sigma-Aldrich Co.) were used to form ZnO microrods. We used Pt paste (PT-1, Dyesol-Timo) as the source for the counter electrode. All the above reagents and materials were used without further purification. 

### 2.2. Preparation of DSSCs

To prepare the working ZnO/FTO electrodes, the FTO glass was cleaned in a detergent solution with sonication for 20 min and then thoroughly rinsed with deionized (DI) water and ethanol. To deposit a seed layer, we spin-coated a solution of Zn(CH_3_COO)_2_·2H_2_O in ethanol (5 mM) on the cleaned FTO glass at 500 rpm for 5 s, at 3000 rpm for 30 s, and at 500 rpm for 5 s. They were then dried on coated glass on a preheated hot plate at 150 °C for 15 min. After this process was repeated three times, the glass was annealed at 350 °C for 15 min. The FTO glass with the seed layer was immersed in an aqueous solution of Zn(NO_3_)_2_·6H_2_O (35 mM) and HMTA (35 mM) at 90 °C for 4–10 h to form ZnO microrods on the FTO substrate. The deposition area was adjusted by masking using Kapton tape. After the deposition, the films were taken out of the growth solution and rinsed with deionized water and ethanol. Subsequently, the ZnO microrods on the FTO glass were annealed at 450 °C for 30 min. Dye-sensitized ZnO/FTO photoelectrodes were prepared through the conventional soaking method, i.e., the ZnO/FTO films were soaked in a 0.5 mM dye (N719) solution (ethanol) for 20 min.

**Figure 1:**
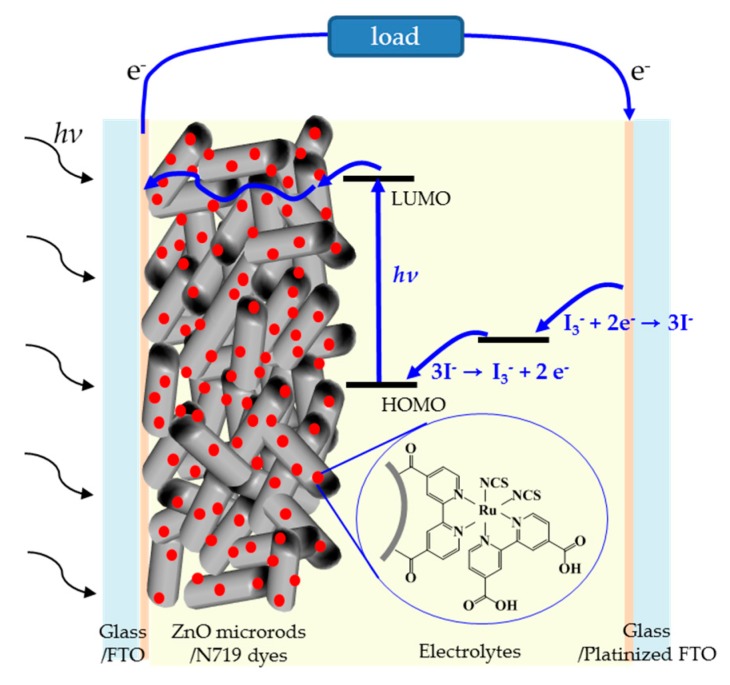
Structure of ZnO-based dye-sensitized solar cells (DSSC) and mechanism for light energy conversion into electric energy.

Two small holes were formed in the FTO glasses and the glasses were thoroughly cleaned with ultrasonification. Pt paste was coated on the cleaned FTO glass via the doctor-blade method and then calcined at 400 °C for 30 min. The resulting counter electrodes and the working ZnO/FTO electrodes were sealed using a 60 μm thick Surlyn. By injecting the electrolyte into the cells through one of the two small holes in the counter electrodes we could prepare ZnO-based DSSCs with a 25 mm^2^ active area. Figure 1 shows a schematic illustration of a ZnO-based DSSC and electron flow when the device is exposed to light.

### 2.3. Measurements 

The morphology of the ZnO microrods was visualized by field emission scanning electron microscopy (FE-SEM; S-4800, Hitachi High-Technology, Tokyo, Japan). A CompactStat potentiostat (Ivium Technologies B.V., Eindhoven, The Netherlands) and a PEC-L01 solar simulator system equipped with a 150 W xenon arc lamp (Peccell Technologies, Inc., Yokohama, Japan) were used to measure the photocurrent and photovoltage. To adjust the light intensity to 1 sun (100 mW/cm^2^) a silicon photodiode (PEC-SI01, Peccell Technologies, Inc., Yokohama, Japan) was utilized. The UV-visible absorbance was measured using a spectrophotometer (NEOSYS 2000, Seoul, SINCO, Korea). An electrochemical analyzer (CompactStat, Ivium Technologies B.V., Eindhoven, The Netherlands) was used for the electrochemical impedance spectroscopic (EIS) analysis. The active area of the ZnO layer was measured using a digital microscope camera (OLYMPUS SZ61) equipped with image analysis software.

## 3. Results and Discussion

### 3.1. Morphological Characteristics of ZnO Layers with Growth Time

Nano- and micro-structured ZnO layers were formed through Equations (1)–(4) on the FTO substrates when the seeded FTO glass was immersed into the growth solution comprised of zinc nitrate and HMTA [(CH_2_)N_4_] at 90 °C. 

(CH_2_)N_4_ (aq) + 6H_2_O (l) → 4NH_3_ (g) + 6HCHO (aq) (1)

NH_3_ (g) + H_2_O (l) → NH_4_^+^ (aq) + OH^−^ (aq) (2)

Zn^2+^ (aq) + 2OH^−^ (aq) → Zn(OH)_2_ (s) (3)

Zn(OH)_2_ (s) → ZnO (s) + H_2_O (l) (4)

HTMA was hydrolyzed, i.e., reacted with water, to yield HCHO and NH_3_. Zn(OH)_2_, precipitated through the reaction between the zinc ions (Zn^2+^) and hydroxide ions (OH^−^) provided from zinc nitrate and ammonium hydroxide (see Equation (2)), respectively, and then dehydrated to form ZnO. Generally, the concentration of the CBD solution determines the density of a nanostructure, while temperature and growth time affect aspect ratio and morphology of the nanostructure [11,12,13,14,15]. 

**Figure 2:**
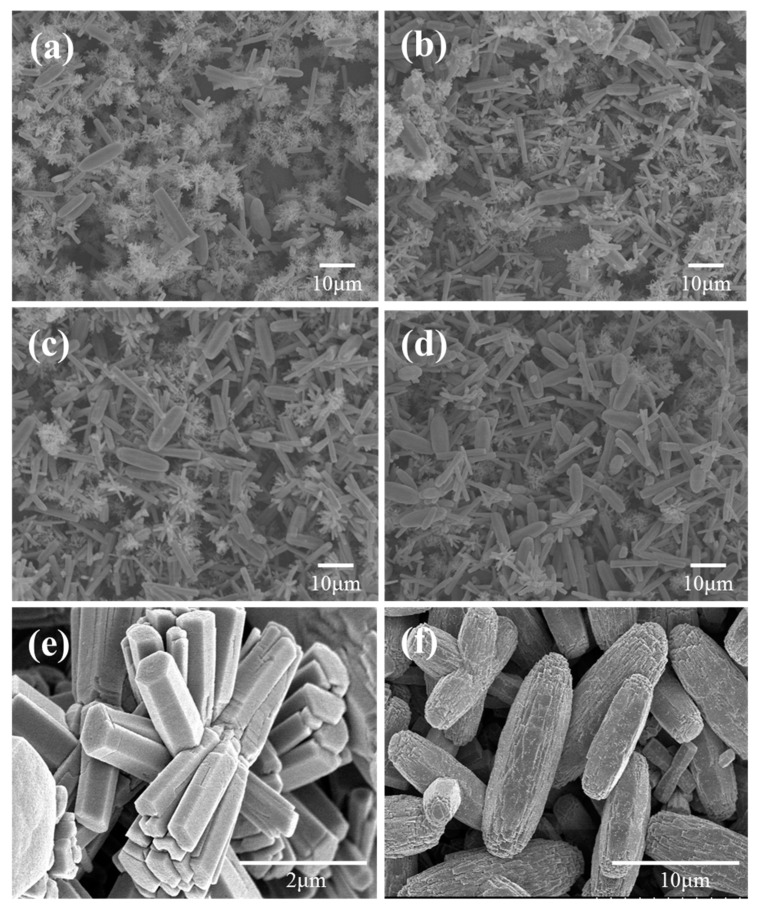
Top-view SEM images of the ZnO layer formed on the FTO glass for (**a**) 4 h, (**b**) 6 h, (**c**) 8 h, and (**d**) 10 h of growth time. Figures (**e**,**f**) are illustrations of nanoflowers and microrods, respectively.

**Figure 3:**
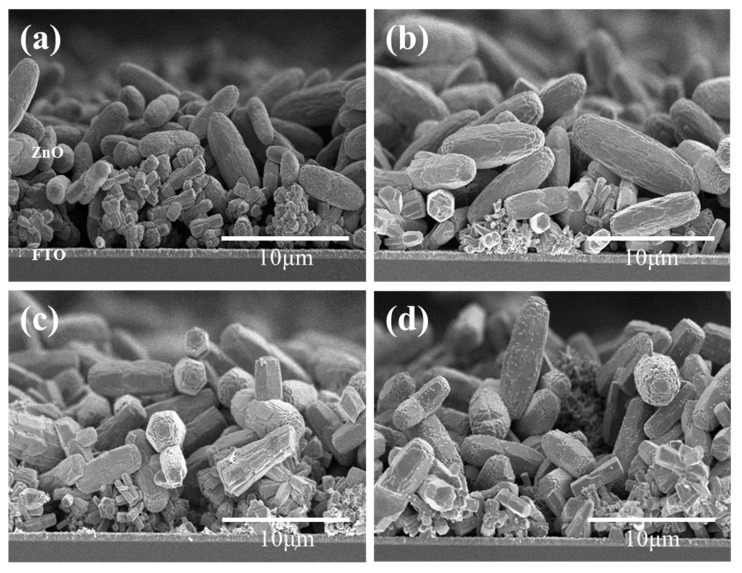
Cross-sectional SEM images of ZnO layer formed on the FTO glass for (**a**) 4 h, (**b**) 6 h, (**c**) 8 h, and (**d**) 10 h growth time.

Using a CBD solution comprising zinc nitrate (35 mM) and HMTA (35 mM), we fabricated a mixed layer of ZnO nanoflowers and microrods at an elevated temperature of 90 °C. Randomly distributed nanoflowers and microrods of ZnO were formed, as illustrated in Figure 2. As a reference, when the growth temperature was lowered to 75 °C, only ZnO nanorods, which were dense and vertically aligned, were formed on the FTO glass (not shown here). As the growth time was extended, the number of nanoflowers was reduced whilst the number of microrods increased. The nanoflowers exhibited a length of approximately 0.54–3.43 μm and a diameter of 350–650 nm. The length and diameter of the microrods were measured to be approximately 6.2–13.1 μm and 2.27–3.55 μm, respectively. A cross-sectional view of the ZnO layers on the FTO glass is shown in Figure 3. ZnO layers composed of nanoflowers and microrods were formed on the FTO glass with thicknesses of about 12, 16, 18, and 19 μm for growth times of 4, 6, 8, and 10 h, respectively.

### 3.2. Photovoltaic Performance of DSSCs with ZnO Nanoflowers and Microrods

**Figure 4:**
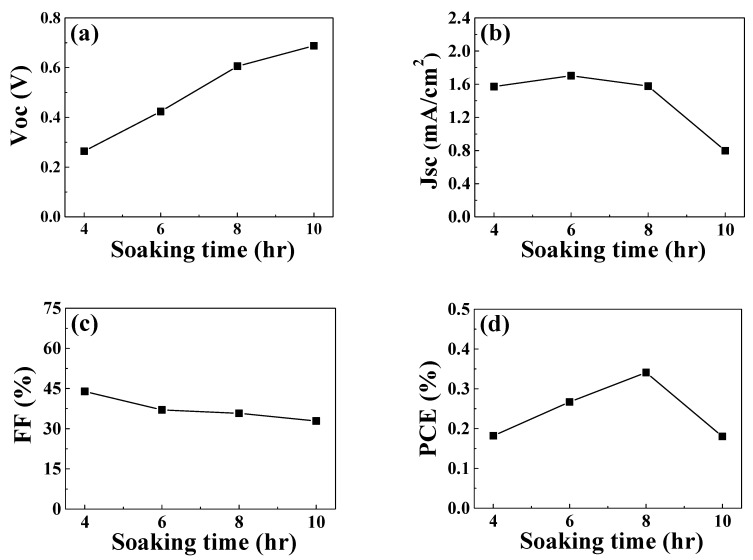
Variation of the photovoltaic performance with growth time: (**a**) *J_sc_*, (**b**) *V_oc_*, (**c**) Fill factor (*FF*), and (**d**) *PCE* of the DSSCs measured under AM 1.5 irradiation.

The ZnO-coated FTO glass was dipped in the ethanolic solution (0.05 mM) of N719 dyes, sensitizing the nanoflowers and microrods and thereby producing sensitized ZnO(4, 6, 8 or 10)/FTO photoelectrodes, where “(4)” indicates that the growth time of ZnO was 4 h. DSSCs were fabricated using the sensitized ZnO/FTO photoelectrodes, and the effects of the growth time on the cell performance were investigated. Four cells were fabricated for each growth time and the PCE of each cell was measured. Among the four cells fabricated for each growth time, one that showed a PCE similar to the averaged value was chosen. Variation curves of cell performance as a function of the growth time are illustrated in Figure 4. Detailed photovoltaic parameters are compared in Figure S1 and Table S1 in the Supplementary Information. Relatively low PCEs were recorded in the DSSCs based on the nanoflowers and microrods. This could be due to the randomly distributed ZnO layers that interrupted the transfer of photo-injected electrons to FTO electrodes compared to those that were vertically aligned. The open-circuit voltage (*V_oc_*) values increased with increased growth time, while the fill factor (*FF*) values were reduced. The *J_sc_* value of the DSSCs remained similar for growth times of 4–8 h, but decreased sharply for 10 h growth time. Overall, the highest PCE value was observed at a growth time of 8 h. In order to explore the higher efficiency of the ZnO(8)/FTO DSSC, we set the ZnO(4)/FTO DSSC as a reference cell. This is because the ZnO(4)/FTO DSSC contains the largest number of nanoflowers among our ZnO/FTO samples.

**Figure 5:**
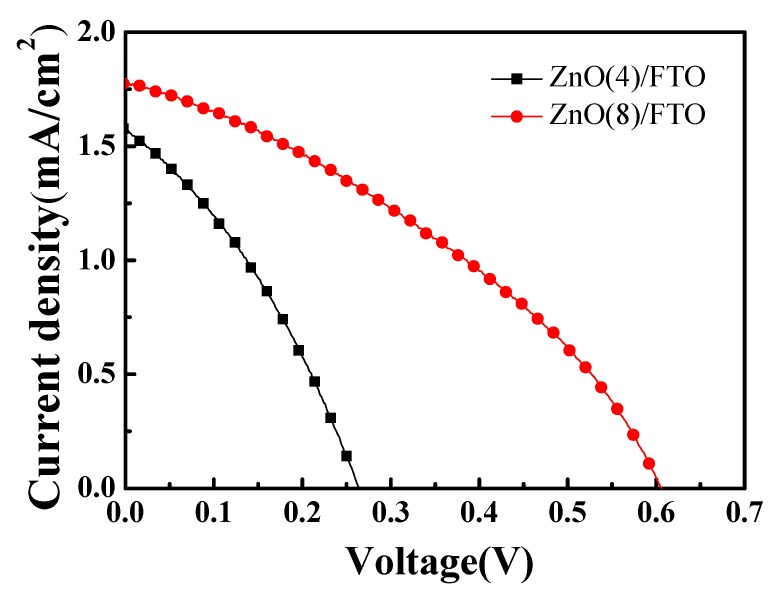
*J*–*V* characteristics of DSSCs with ZnO(4)/FTO and ZnO(8)/FTO photoelectrodes.

Figure 5 presents the current density (*J*) and voltage (*V*) curves of the ZnO(8)/FTO and the ZnO(4)/FTO DSSCs. The DSSC performance is compared in Table 1. The DSSC with the ZnO(8)/FTO photoelectrode showed a PCE of 0.341%, while the reference cell with the ZnO(4)/FTO showed a PCE of 0.182%. The improved PCE was mostly due to the increment of the *V_oc_*. A significant increase in *V_oc_* of the ZnO(8)/FTO cell is notable compared to the reference cell. Under constant illumination, the *V_oc_* value of DSSCs can be expressed as a function of the quasi-Fermi level (*E_Fn_*) and to the dark value (*E_F0_*). This is related to the thermal energy (*k_B_T*; 4.11 × 10^−21^ J at 25 °C), the Boltzmann constant (*k_B_*), the absolute temperature (*T*), the positive elementary charge (*e*; 1.602 × 10^−19^ C), the concentration in the dark (*n_0_*), and the free electron density in the ZnO photoelectrode (*n*), as represented in Equation (5) [16,17,18]:

*V_oc_* = (*E_Fn_* − *E_F0_*)/*e* = (*k_B_T*/*e*)·ln(*n*/n_0_) (5)

It can be seen from Equation (5) that *V_oc_* and *n* are closely correlated. In addition, *n* is affected by both the electron injection from the photoexcited dyes to the ZnO conduction bands and the charge recombination between photoinjected electrons and triiodide anion in the electrolyte. Therefore, higher electron injection and lesser back electron transfer is necessary to increase *n* and thereby to improve *V_oc_*. To estimate electron injection in the ZnO/FTO photoelectrodes, we measured amounts of N719 dyes adsorbed on the surface of ZnO nanoflowers and microrods using the Beer–Lambert equation [19,20,21]. The adsorbed amounts of dyes, which were measured using 5 cells, for the ZnO(8)/FTO and the ZnO(4)/FTO photoelectrodes averaged 2.43 × 10^−5^ and 6.33 × 10^−5^ mol/cm^3^, respectively. About a 60% reduction in the dye-loading amount was observed in the ZnO(8)–ZnO/FTO compared to the ZnO(4)/FTO. A decrease in the adsorption of N719 dyes on the photoelectrodes could lead to generating fewer electrons and holes, reducing the total number of photoinjected electrons in the device and thus lowering the *n* value. 

**Figure 6:**
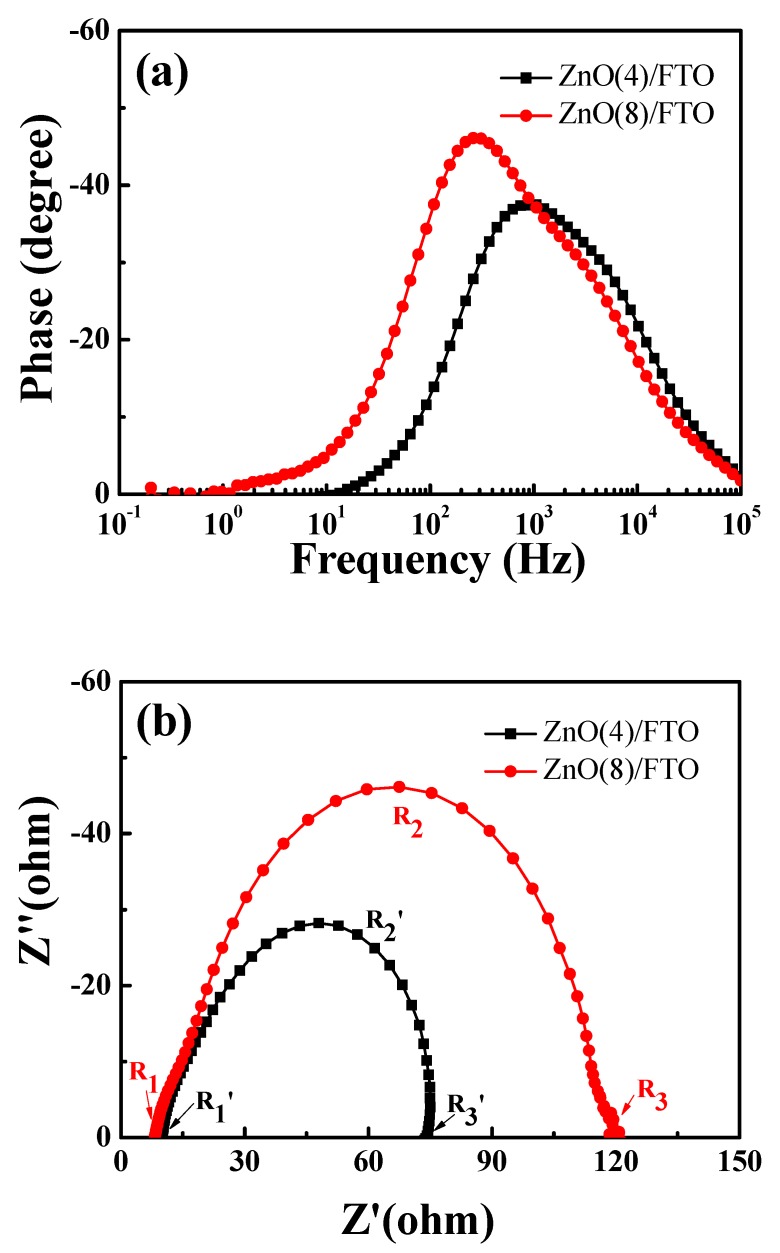
Electrochemical impedance spectroscopic (EIS) spectra of DSSCs with the ZnO(4)/FTO and the ZnO(8)/FTO photoelectrodes; (**a**) Bode and (**b**) Nyquist plots measured at −0.7 V in the dark.

The reduced charge recombination on the interface of the nano- and micro-structured ZnO and electrolyte increased the *n* value and thereby the *V_oc_* value. To confirm this, we conducted EIS measurements. EIS is a powerful tool for investigating the charge transport kinetics and the charge recombination in DSSCs [22,23,24,25]. The results measured at −0.7 V in the dark are shown in Figure S2 in the Supplementary Information section. The Bode phase plots of the EIS spectra for the DSSCs with the ZnO(4)/FTO and the ZnO(8)/FTO electrodes are displayed in Figure 6a. The EIS Bode phase plots of the DSSCs with the ZnO(4)/FTO and the ZnO(8)/FTO gave peak frequencies (f_max_) of 1060 and 277 Hz, respectively. The electron lifetime estimated from the equation (τ_n_ = 1/2πf_max_) [23,24] was determined to be 0.150 and 0.575 ms for the DSSCs with the ZnO(4)/FTO and the ZnO(8)/FTO photoelectrodes, respectively. A prolonged lifetime (an approximately 380% increase) of the electrons injected to ZnO from the dyes was obtained for the ZnO(8)/FTO cell, as compared to that of the reference device. The Nyquist plots of the EIS spectra for the DSSCs are presented in Figure 6b. The Nyquist plots typically include three semicircles: the first semicircle (R_1_) corresponds to the redox reaction at the Pt counter electrode, the second one (R_2_) corresponds to the electron transfer at the ZnO/dye/electrolyte interface, and the third one (R_1_) corresponds to the carrier transport by ions in the electrolytes [24,25,26]. It was noted that by the extended growth time the impedance component of R_2_ in the medium-frequency region was increased. The larger semicircle of R_2_ indicates a more delayed charge recombination at the dye-sensitized ZnO/electrolyte interface [24,27]. This can be elucidated by the reduced surface area of the ZnO(8)/FTO, as compared in Figure 2. In other words, by extending the growth time from 4 to 8 h, the number of nanoflowers (high surface area) was decreased, whilst the number of microrods (relatively low surface area) was increased. The decreased surface area induced a reduction of contact area between ZnO layers and electrolytes, retarding the charge recombination reaction [2e^−^ (ZnO) + I_3_^−^ → 3I^−^]. 

**Figure 7:**
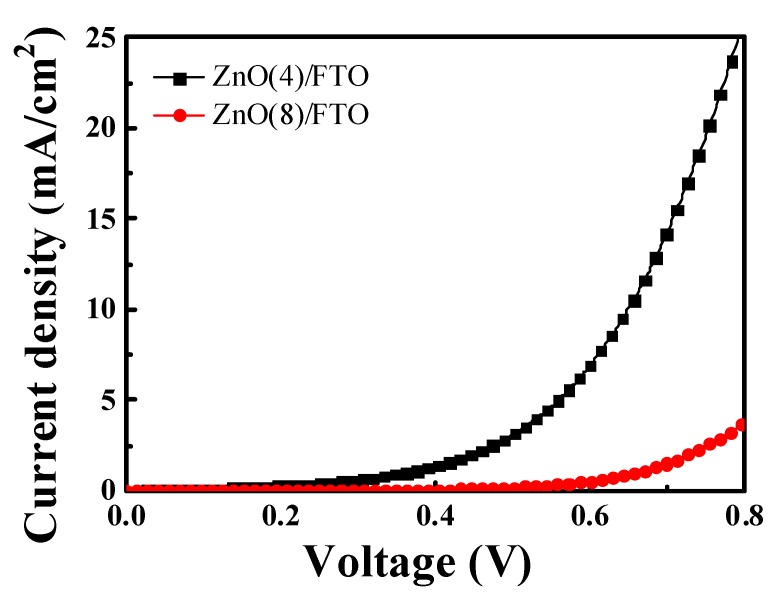
Dark current-voltage characteristics of DSSCs with ZnO(4)/FTO and ZnO(8)/FTO photoelectrodes.

The dark currents can be utilized to evaluate the interfacial charge recombination in DSSCs [27,28,29]. Increased growth time decreased the dark currents, as illustrated in Figure S3 in the Supplementary Information section. Figure 7 shows the dark currents of the ZnO(4)/FTO and the ZnO(8)/FTO DSSCs as a function of the applied potential. Throughout the range of measured potential, the dark currents of the ZnO(8)/FTO DSSC were lower than those of the reference cell. During measurements of dark currents, electrons injected from FTO to the ZnO conduction band transferred through the ZnO layer and then recombined with electrolytes. The lowered current density in the device with the ZnO(8)/FTO thus indicated that the charge recombination rate between injected electrons and triiodide ions was retarded compared with the rate of the reference cell with the ZnO(4)/FTO. The observation of the retarded charge recombination was consistent with the results of the EIS measurements. The prolonged electron lifetime (Bode plots) and the retardation of interfacial charge recombination (Nyquist plots and dark currents) thus indicated that the *n* value was increased with extended growth time and thereby the *V_oc_* value.

The *J_sc_* value of the ZnO(8)/FTO DSSC (1.577 mA/cm^2^) is very similar to that of the reference device with the ZnO(4)/FTO (1.572 mA/cm^2^). The *J_sc_* value is determined by Equation (6) [5,6]:

*J_sc_* = ∫LHE(λ)·η_inj_·η_col_ dλ (6)

where LHE is the light harvesting efficiency of dyes at a given wavelength(λ), η_inj_ is the electron injection efficiency from excited dyes, and η_coll_ is the electron collection efficiencies of the photoinjected electrons to the FTO electrode.

The LHE is related to the light absorbance (A) of the dyes adsorbed on the ZnO surface, i.e., LHE = 1 − 10^−A^ [30,31]. Thus, the amount of adsorbed dyes should be measured to investigate the effects of the LHE on the *J_sc_* value. As mentioned previously, the amount of N719 dyes adsorbed on the ZnO(8)/FTO decreased by approximately 60%, compared to the ZnO(4)/FTO, due to the decreased surface area of ZnO. This indicates that extended growth time could decrease the LHE.

The value of η_col_ depends on the interfacial charge recombination of photoinjected electrons. In other words, more electrons can be collected on the transparent FTO electrode by reducing the charge recombination. As discussed above, EIS and dark current measurements show that by extending the growth time from 4 to 8 h, the lifetime of photoinjected electrons was prolonged and the charge recombination was retarded. This was favorable for increasing the η_coll_ value. Therefore, the *J_sc_* value of the cell with the ZnO(8)/FTO was similar to that of the reference device because the decrease in the LHE was offset by an increase in the η_col_. 

By extending the growth time, the *FF* value of the DSSC decreased to 35.77% from 43.94% of the reference device. Generally, the *FF* value is influenced by the shunt (*R_sh_*) and series (*R_se_*) resistances. In other words, an increase of *R_sh_* and a reduction of *R_se_*, or both, could improve the *FF* value [32,33,34]. The *R_sh_* and *R_se_* values were acquired from the slope of the *J*–*V* curves at *J_sc_* and *V_oc_*, respectively [32,33], as compared in Table 1. The *R_sh_* value of the DSSC with the ZnO(8)/FTO improved by 973.1 Ωcm^2^ compared to that of the reference cell (345.7 Ωcm^2^). The increase in *R_sh_* can be explained by the delayed charge recombination between photo-injected electrons and triiodide ions in the device with the ZnO(8)/FTO [33,34], as demonstrated in both the EIS and the dark current measurements. Meanwhile, the *R_se_* value of the cell with the ZnO(8)/FTO (131.4 Ωcm^2^) was measured to be higher than that of the reference device (97.8 Ωcm^2^). This can be better explained by the larger gaps between ZnO microrods in the ZnO(8)/FTO than those between nanoflowers in the ZnO(4)/FTO, causing interruption of electron transport. Thus, the elevation of *R_se_*, as compared to the reference device, can be attributed to the decrease in the *FF* value of the ZnO(8)/FTO DSSC. 

## 4. Conclusions

ZnO nanoflowers and microrods fabricated by CBD at 90 °C were sensitized with N719 dyes and the resulting films were employed for photoanodes of DSSCs. The ZnO(8)/FTO DSSC (microrod-rich) showed a significant increase in the *V_oc_* resulting in a PCE of 0.341% (*V_oc_*= 0.606 V, *J_sc_*= 1.577 mA/cm^2^, and *FF* = 35.77%), as compared to those of the reference ZnO(4)/FTO DSSC (nanoflower-rich) (PCE = 0.182%; *V_oc_*= 0.264 V, *J_sc_*= 1.572 mA/cm^2^, and *FF* = 43.94%). This increase in the *V_oc_*, and thereby the PCE, was attributed to a retardation of the interfacial charge recombination. This study therefore suggests that the growth temperature and time of ZnO determine the nanostructure and morphology and ZnO microrods improve the PCEs of ZnO-based DSSCs more effectively than ZnO nanoflowers.

## Figures and Tables

**Table 1:** Photovoltaic properties of DSSCs with ZnO(4)/FTO and ZnO(8)/FTO photoelectrodes.

Applied Photoelectrodes	*V_oc_* (mV)	*J_sc_* (mA/cm^2^)	*FF* (%)	*η* (%)	*R_se_* (Ωcm^2^)	*R_sh_* (Ωcm^2^)
ZnO(4)/FTO	0.264	1.572	43.94	0.182	97.8	345.7
ZnO(8)/FTO	0.606	1.577	35.77	0.341	131.4	973.1

## Supplementary Materials

The following are available online at https://www.mdpi.com/2079-4991/9/12/1645/s1, Figure S1: *J*–*V* characteristics of DSSCs with ZnO/FTO photoelectrodes, Table S1: Photovoltaic parameters of DSSCs with ZnO/FTO photoelectrodes, Figure S2: EIS spectra of the DSSCs with the ZnO/FTO photoelectrodes; (a) Bode and (b) Nyquist plots measured at −0.7 V in the dark, Figure S3: Dark current–voltage characteristics of DSSCs with ZnO/FTO photoelectrodes.
