# Supplementary material for: Photovoltaic Performance of Dye-Sensitized Solar Cells Containing ZnO Microrods
Source: Nanomaterials (Basel). 2019 Nov 20;9(12):1645. doi: 10.3390/nano9121645 (PMC6956303; doi:10.3390/nano9121645)
Supplement: Supplementary file 1 [file nanomaterials-09-01645-s001.pdf]

nanomaterials

MDPI

Article

Photovoltaic Performance of Dye-Sensitized Solar Cells Containing ZnO Microrods

Seong Il Cho [1,†], Hye Kyeong Sung [2,†], Sang-Ju Lee [3], Wook Hyun Kim [3], Dae-Hwan Kim [3,*] and Yoon Soo Han [1,*]

[1] School of Advanced Materials and Chemical Engineering, Daegu Catholic University, Gyeongbuk 38430, Korea; pokw1231@naver.com
[2] Department of Organic Material Science and Engineering, Pusan National University, Busan 46241, Korea; amrm1995@naver.com
[3] Division of Energy Technology, Daegu Gyeongbuk Institute of Science and Technology (DGIST), Daegu 42988, Korea; tglee@dgist.ac.kr (S.-J.L.); kwh1980@dgist.ac.kr (W.H.K.)
[*] Correspondence: monolith@dgist.ac.kr (D.-H.K.); yshancu@cu.ac.kr (Y.S.H.); Tel: +82-53-785-3720 (D.-H.K.); +82-53-850-2773 (Y.S.H.); Fax: +82-53-785-3739 (D.-H.K.); +82-53-359-6662 (Y.S.H.)
[†] These authors contributed equally to this work.

1. Current density-voltage profiles of DSSCs with ZnO/FTO photoelectrodes

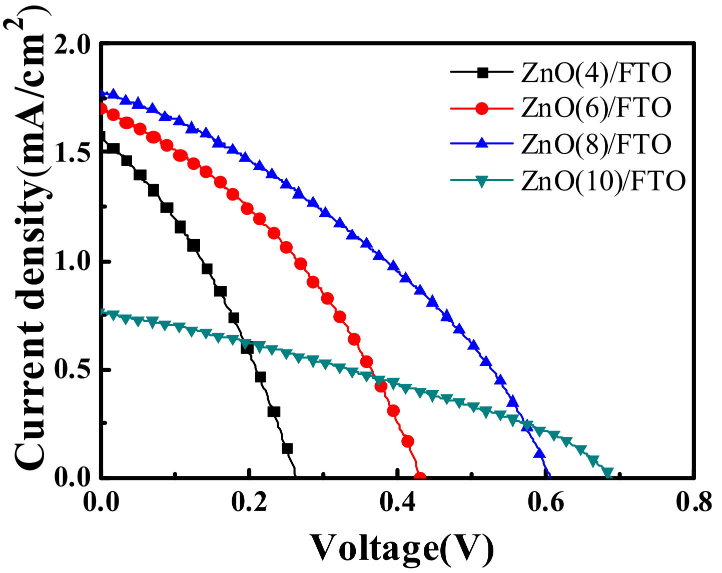

Figure S1. *J*–*V* characteristics of DSSCs with ZnO/FTO photoelectrodes.

Table S1. Photovoltaic parameters of DSSCs with ZnO/FTO photoelectrodes

Applied photoelectrodes	V_{oc} (mV)	J_{sc} (mA/cm^2)	*FF* (%)	η (%)

ZnO(4)/FTO	0.264	1.572	43.94	0.182
ZnO(6)/FTO	0.424	1.703	37.02	0.267
ZnO(8)/FTO	0.606	1.577	35.77	0.341
ZnO(10)/FTO	0.688	1.799	32.88	0.180

2. Electrochemical impedance spectroscopic analysis of DSSCs with ZnO/FTO photoelectrodes

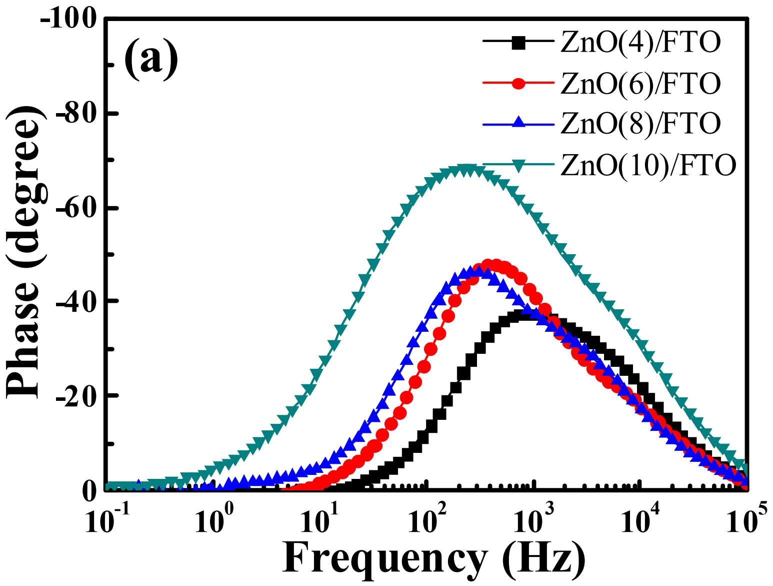

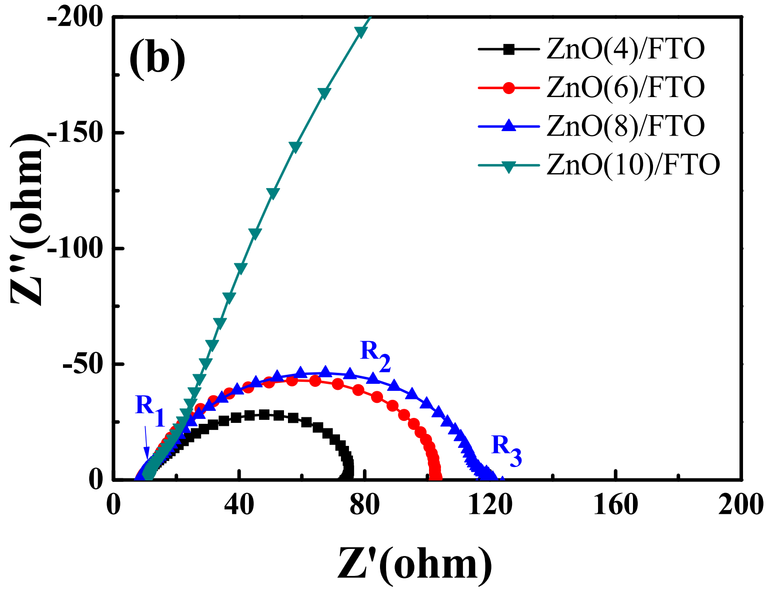

Figure S2. EIS spectra of the DSSCs with the ZnO/FTO photoelectrodes; (**a**) Bode and (**b**) Nyquist plots measured at −0.7 V in the dark.

3. Dark current-voltage profiles of DSSCs with ZnO/FTO photoelectrodes

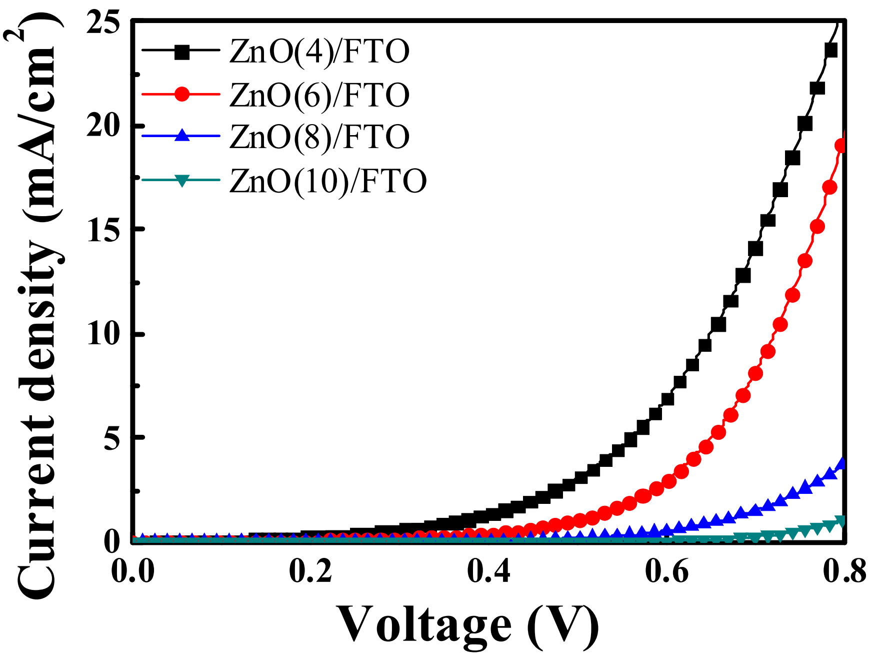

Figure S3. Dark current–voltage characteristics of DSSCs with ZnO/FTO photoelectrodes.
